# Supplementary material for: Effect of the Morphology and Electrical Property of Metal-Deposited ZnO Nanostructures on CO Gas Sensitivity
Source: Nanomaterials (Basel). 2020 Oct 27;10(11):2124. doi: 10.3390/nano10112124 (PMC7692783; doi:10.3390/nano10112124)
Supplement: Supplementary file 1 [file nanomaterials-10-02124-s001.pdf]

Supporting information

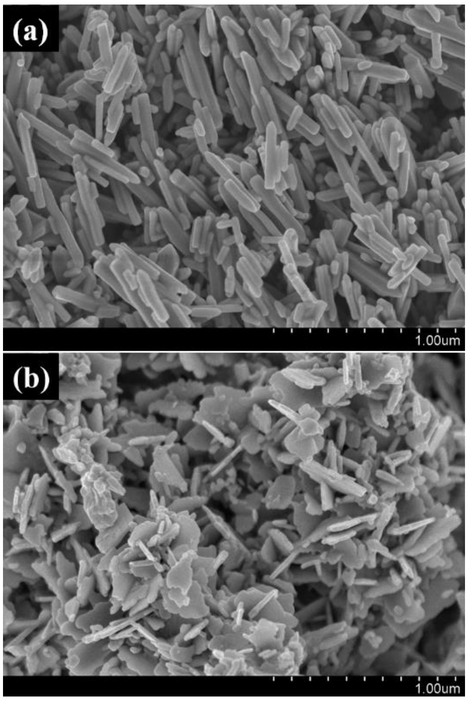

Figure S1. SEM image of ZnO nanostructures (**a**) ZnO nanorod (**b**) ZnO nanoplate

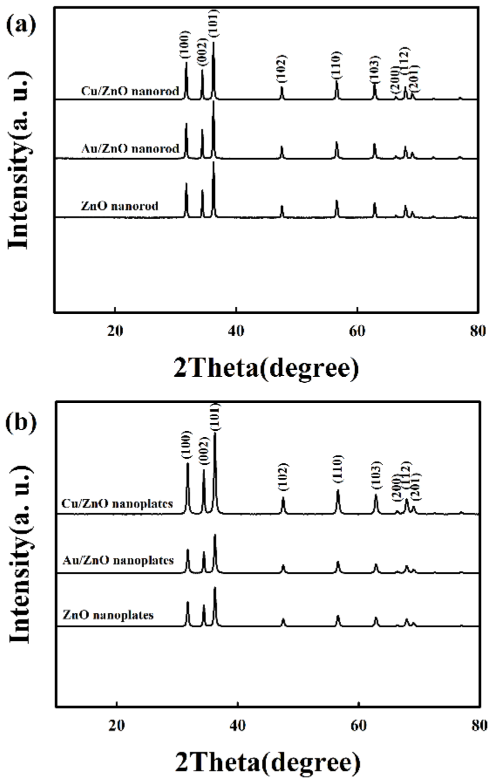

Figure S2. XRD patterns of the samples
